# Supplementary material for: Prosocial behavior, social reward and affective state discrimination in adult male and female mice
Source: Sci Rep. 2023 Apr 5;13:5583. doi: 10.1038/s41598-023-32682-6 (PMC10076499; doi:10.1038/s41598-023-32682-6)
Supplement: Supplementary file 1 — Supplementary Information. [file 41598_2023_32682_MOESM1_ESM.docx]

**Supplementary Information**

**Prosocial behavior, social reward and affective state discrimination in adult male and female mice**

Klaudia Misiołek, Marta Klimczak, Magdalena Chrószcz, Łukasz Szumiec, Anna Bryksa#, Karolina Przyborowicz, Jan Rodriguez Parkitna*, Zofia Harda*

Department of Molecular Neuropharmacology, Maj Institute of Pharmacology of the Polish Academy of Sciences, Krakow, Poland

*Corresponding authors:

Zofia Harda, harda@if-pan.krakow.pl

Jan Rodriguez Parkitna, janrod@if-pan.krakow.pl

Department of Molecular Neuropharmacology, Maj Institute of Pharmacology of the Polish Academy of Sciences, Smętna 12, 31-343 Krakow, Poland

# Present address: Laboratory of Emotions Neurobiology, Nencki Institute of Experimental Biology, 3 Pasteur Street, 02-093 Warszawa, Poland

**
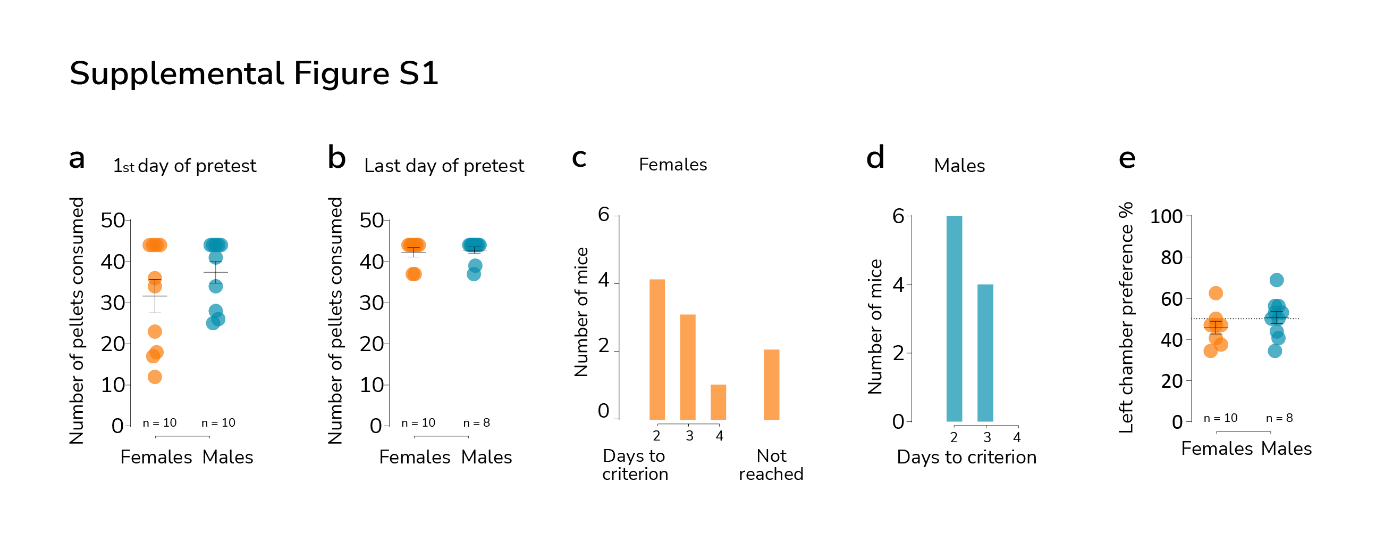
Supplementary Figures**

**
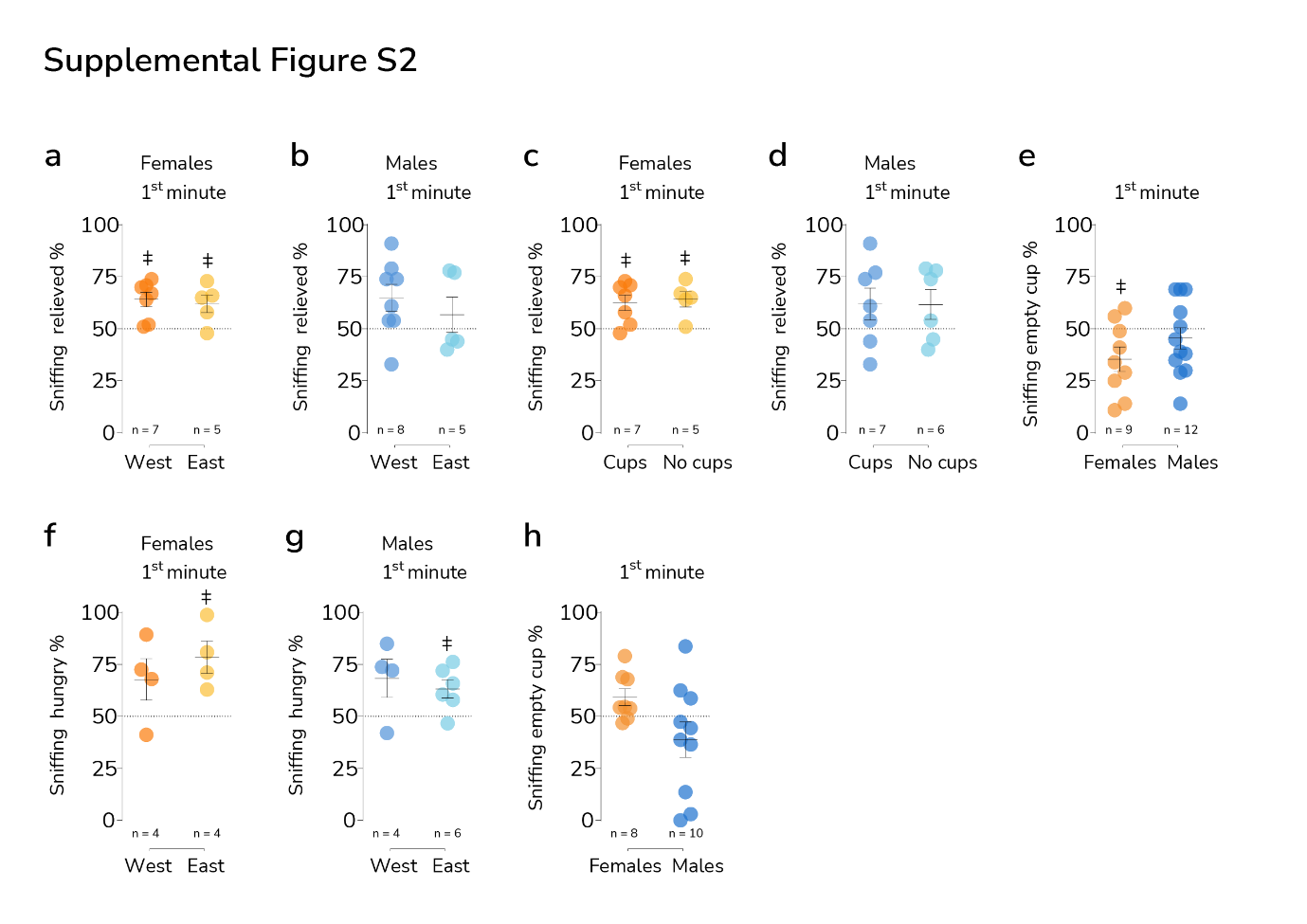
Rewards consumed during the pretest phase of the prosocial task.** (**a-b**) The number of food pellets consumed by the female and male actors during the first and final days of the pretest, respectively. Each point represents an individual female or male mouse, with the mean and s.e.m. shown in black and the group sizes indicated below. (**c-d**) The number of female and male mice that reached the inclusion criterion of 37 out of 44 food pellets consumed on two consecutive sessions. As shown in (**c**), two female animals did not meet the required criterion. (**e**) No differences form chance level in average preference for the left chamber of the testing apparatus during pretest in female and male mice, respectively.

**Context effects in the affective state determination task.** (**a-b**) No effect of the position of the relieved demonstrator (West or East) on the proportion of the time the observer spent sniffing him in female and male mice, respectively. A significant preference (greater than 0) is indicated by a “‡” (one-sample t-test p<0.05) (**c-d**) No effect of the presence of the cups during habituation. (**e**) Final day of habituation. No effect of sex on time spent sniffing the empty cup in which the relieved demonstrator was placed during the test. Recordings of final day of habituation of 3 females and 1 male have been lost. (**f**-**g**) No effect of the position of the hungry demonstrator (West or East) on the proportion of the time the observer spent sniffing him in female and male mice, respectively. (**h**) Final day of habituation. No effect of sex on time spent sniffing the empty cup in which the hungry demonstrator was placed during the test.

**Supplementary Tables**

| **Table S1. Prosocial choice test.** Number of mice excluded based on predefined criteria**.** | | | | | | | | | | |
| --- | --- | --- | --- | --- | --- | --- | --- | --- | --- | --- |
| Sex | Initial n | Number of mice that did not meet criterion | | Tested n |  |  |  |  |  |  |
|  |  | criterion A | criterion B |  |  |  |  |  |  |  |
| females | 10 | 2 | 0 | 8 |  |  |  |  |  |  |
| males | 10 | 0 | 0 | 10 |  |  |  |  |  |  |
|  |  |  |  |  |  |  |  |  |  |  |
| Criterion A: two days in a row consumed >84% of the reward pellets delivered during the pretest session (37 out of 44)  Criterion B: average pretest side preference below 70% | | | | | | | | | |  |
|  | | | |  |  |  |  |  |  |  |

| **Table S2. Prosocial choice test.** Average pretest and test preference for prosocial compartment. | | | | | | | | | | | | | | | | | |
| --- | --- | --- | --- | --- | --- | --- | --- | --- | --- | --- | --- | --- | --- | --- | --- | --- | --- |
| Fig. | Sex | n | Preference during pretest % | |  | Preference during test % | | Mean difference | Paired Student’s t-test | | |  | | |  |  |  |
|  |  |  | M | SEM |  | M | SEM |  | t | df | p |  |  |  |  |  |  |
| 1e | females | 8 | 44,10 | 2,70 |  | 54,30 | 3,00 | 10,16 | 4,33 | 7,00 | **0,00** |  | | |  |  |  |
| 1f | males | 10 | 47,50 | 2,90 |  | 47,80 | 1,50 | 0,31 | 0,10 | 9,00 | 0,92 |  |  |  |  |  |  |

| **Table S3. Prosocial choice test.** Prosociality score. Females and males comparison. | | | | | | | | | | | | | | | |  | |  |
| --- | --- | --- | --- | --- | --- | --- | --- | --- | --- | --- | --- | --- | --- | --- | --- | --- | --- | --- |
| Fig. | Sex | Prosociality score %^a^ | | | | | | | | | | | | |  | | | |
|  |  | n | M | SEM |  | One sample t test. Comparison with hypothetical mean (0) | | |  | Mean difference |  | Unpaired Student’s t-test | | |  | |  | |
|  |  |  |  |  |  | t | df | p |  |  |  | t | df | p |  | |  | |
| 1g | females | 8 | 10,20 | 2,30 |  | 4,35 | 7 | **0,00** |  | 9,88 |  | 2,47 | 16,00 | **0,03** |  | |  | |
|  | males | 10 | 0,30 | 3,00 |  | 0,10 | 9 | 0,92 |  |  |  |  |  |  |  |  |  |  |
| ^a^ Mean % of prosocial choices of the last two days of pretest subtracted from mean % of prosocial choices of 4 days of test. | | | | | | | | | | | | | | |  | | | |

| **Table S2.** Continued. Average preference for the left chamber of the testing apparatus during pretest. | | | | | | | | | | | | | | |
| --- | --- | --- | --- | --- | --- | --- | --- | --- | --- | --- | --- | --- | --- | --- |
| Fig. | Sex | n | M | SEM |  | One sample t test. Comparison with hypothetical mean (50%) | | |  | Mean difference |  | Unpaired Student’s t-test | | |
|  |  |  |  |  |  | t | df | p |  |  |  | t | df | p |
| S1e | females | 8 | 45,71 | 3,07 |  | 1,40 | 7,00 | 0,20 |  | 4,93 |  | 1,13 | 16,00 | 0,27 |
|  | males | 10 | 50,64 | 3,015 |  | 0,21 | 9,00 | 0,84 |  |  |  |  |  |  |

| **Table S4.** Descriptive Statistics and Correlations. | | | | | | | | | | | | | | | | | |  |
| --- | --- | --- | --- | --- | --- | --- | --- | --- | --- | --- | --- | --- | --- | --- | --- | --- | --- | --- |
| Sex | Behavioral test | Experiment variant | Variable | n | M | SEM | Subject's weight [g]**^a^** | | |  | Stimulus' weight [g]**^b^** | | |  | Weight difference %**^c^** | | |  |
|  |  |  |  |  |  |  | r^2^ | r | p |  | r^2^ | r | p |  | r^2^ | r | p |  |
| females | Prosocial choice test**^1^** |  | Prosociality score % | 8 | 10,18 | 2,34 | 0,00 | 0,06 | 0,88 |  | 0,09 | 0,30 | 0,46 |  | 0,05 | -0,22 | 0,59 |  |
|  | Affective state discrimination test**^2^** | relieved | Sniffing relieved % | 12 | 63,83 | 2,72 | 0,40 | -0,62 | **0,02** |  | 0,50 | -0,70 | **0,00** |  | 0,28 | 0,53 | 0,07 |  |
|  |  | hungry | Sniffing hungry % | 8 | 73,10 | 6,18 | 0,19 | -0,44 | 0,28 |  | 0,00 | 0,04 | 0,92 |  | 0,48 | -0,69 | 0,06 |  |
|  | Social conditioned place preference test**^3^** |  | Preference score [s] | 16 | 285,80 | 87,07 | 0,09 | 0,31 | 0,24 |  | NA | NA | NA |  | NA | NA | NA |  |
| males | Prosocial choice test**^1^** |  | Prosociality score % | 10 | 0,30 | 3,03 | 0,13 | -0,36 | 0,38 |  | 0,09 | 0,30 | 0,30 |  | 0,54 | -0,73 | **0,01** |  |
|  | Affective state discrimination test**^2^** | relieved | Sniffing relieved % | 13 | 62,15 | 4,96 | 0,07 | 0,26 | 0,37 |  | 0,05 | -0,24 | 0,42 |  | 0,00 | 0,02 | 0,93 |  |
|  |  | hungry | Sniffing hungry % | 10 | 65,22 | 4,26 | 0,00 | -0,03 | 0,93 |  | 0,04 | -0,19 | 0,59 |  | 0,09 | 0,30 | 0,39 |  |
|  | Social conditioned place preference test**^3^** |  | Preference score [s] | 12 | 221,60 | 90,59 | 0,04 | -0,12 | 0,54 |  | NA | NA | NA |  | NA | NA | NA |  |
| \| **Table S5. Social conditioned place preference test.** Average pretest and test preference for social context. \| \| \| \| \| \| \| \| \| \| \| \| \| \| \| \| \| \| \| --- \| --- \| --- \| --- \| --- \| --- \| --- \| --- \| --- \| --- \| --- \| --- \| --- \| --- \| --- \| --- \| --- \| --- \| \| Fig. \| Sex \| n \| Preference during pretest [s] \| \|  \| Preference during test [s] \| \| Mean difference \| Paired Student’s t-test \| \| \| \| M \| SEM \| M \| SEM \| t \| df \| p \| \| 2a \| females \| 16 \| 893,70 \| 34,85 \|  \| 1040,00 \| 43,56 \| 146,30 \| 2,83 \| 15,00 \| **0,01** \| \| 2b \| males \| 12 \| 855,80 \| 40,59 \|  \| 1010,00 \| 45,28 \| 154,20 \| 4,20 \| 11,00 \| **0,00** \| \| 2d \| males \| 8 \| 918,90 \| 26,18 \|  \| 838,20 \| 58,76 \| 80,69 \| 1,18 \| 7,00 \| 0,28 \| \|  \|  \|  \|  \|  \|  \|  \|  \|  \|  \|  \|  \|  \|  \|  \|  \|  \|   **1a.** Actor's weight on the first day of test; **1b.** Partner's weight on the first day of test; **1c.** Percentage of weight difference between actor and partner on the first day of test. 2a. Observer's weight on the first day of adaptation; 2b. Relieved/hungry demonstrator's weight on the first day of adaptation; 2c. Percentage of weight difference between observer and relieved/hungry demonstrator on the first day of adaptation. 3a. Subject's weight on the day of posttest. | | | | | | | | | | | | | | | | | |  |
|  |  |  |  |  |  |  |  |  |  |  |  |  |  |  |  |  |  |  |

| **Table S6. Social conditioned place preference test.** Preference score. Females and males comparison. | | | | | | | | | | | | | | | | | |
| --- | --- | --- | --- | --- | --- | --- | --- | --- | --- | --- | --- | --- | --- | --- | --- | --- | --- |
| Fig. | Sex | Social preference score [s]^a^ | | | | | | | | | | | | |  | |  |
|  |  | n | M | SEM |  | One sample t test. Comparison with hypothetical mean (0) | | |  | Mean difference |  | Unpaired Student’s t-test | | |  |  |  |
|  |  |  |  |  |  | t | df | p |  |  |  | t | df | p |  |  |  |
| 2c | females | 16 | 285,80 | 87,07 |  | 3,28 | 15,00 | **0,01** |  | 64,20 |  | 0,50 | 26,00 | 0,62 |  |  |  |
|  | males | 12 | 221,60 | 90,59 |  | 2,45 | 11,00 | **0,03** |  |  |  |  |  |  |  |  |  |
| 2d | males | 8 | -119,40 | 117,70 |  | 1,02 | 7,00 | 0,34 |  |  |  |  |  |  |  |  |  |
| ^a^ Time spent in social context pretest [s] subtracted from time spent in social context posttest [s] | | | | | | | | | | | | | | |  | |  |

| **Table S7. Affective state discrimination test.** Anova with repeated measures table for sniffing demonstrators [s] | | | | | | | | | | | | | | | | | | | | | | | |
| --- | --- | --- | --- | --- | --- | --- | --- | --- | --- | --- | --- | --- | --- | --- | --- | --- | --- | --- | --- | --- | --- | --- | --- |
|  |  |  |  |  |  |  |  |  |  |  |  |  |  |  |  |  |  |  |  |  |  |  |  |
| Demonstrators state | Fig. | Sex | Source of variation | | | | | | | | | | | | | | | | | |  |  |  |
|  |  |  | Time | | | | |  | Demonstrator's state | | | | |  | Interaction | | | | | |  |  |  |
|  |  |  | SS | DF | MS | F | p |  | SS | DF | MS | F | p |  | SS | DF | MS | F | p |  |  |  |  |
| relieved | 3b | females | 315,50 | 3,00 | 105,20 | 3,68 | **0,02** |  | 106,90 | 1,00 | 106,90 | 2,33 | 0,14 |  | 208,30 | 3,00 | 69,44 | 2,43 | 0,07 |  |  |  |  |
|  |  |  |  |  |  |  |  |  |  |  |  |  |  |  |  |  |  |  |  |  |  |  |  |
|  |  |  |  |  |  |  |  |  |  |  |  |  |  |  |  |  |  |  |  |  |  |  |  |
|  | 3c | males | 129,50 | 3,00 | 43,16 | 1,25 | 0,30 |  | 584,30 | 1,00 | 584,30 | 6,23 | **0,02** |  | 185,90 | 3,00 | 61,98 | 1,80 | 0,15 |  |  |  |  |
|  |  |  |  |  |  |  |  |  |  |  |  |  |  |  |  |  |  |  |  |  |  |  |  |
|  |  |  |  |  |  |  |  |  |  |  |  |  |  |  |  |  |  |  |  |  |  |  |  |
|  |  |  |  |  |  |  |  |  |  |  |  |  |  |  |  |  |  |  |  |  |  |  |  |
| hungry | 3e | females | 49,48 | 3,00 | 16,49 | 0,60 | 0,57 |  | 370,30 | 1,00 | 370,30 | 5,75 | **0,03** |  | 425,00 | 3,00 | 141,70 | 5,16 | **0,00** |  |  |  |  |
|  |  |  |  |  |  |  |  |  |  |  |  |  |  |  |  |  |  |  |  |  |  |  |  |
|  |  |  |  |  |  |  |  |  |  |  |  |  |  |  |  |  |  |  |  |  |  |  |  |
|  | 3f | males | 323,10 | 3,00 | 107,70 | 5,53 | **0,00** |  | 45,52 | 1,00 | 45,52 | 0,80 | 0,38 |  | 314,80 | 3,00 | 104,90 | 5,39 | **0,00** |  |  |  |  |
|  |  |  |  |  |  |  |  |  |  |  |  |  |  |  |  |  |  |  |  |  |  |  |  |

| **Table S7.** Continued. | | |  |  |  |  |  |  |  |  |  |
| --- | --- | --- | --- | --- | --- | --- | --- | --- | --- | --- | --- |
| Demonstrators state | Fig. | Sex | Comparison of time sniffing relieved vs neutral demonstrators [s] | | | | | | | |  |
|  |  |  | Time [minute] | M_relieved_ | M_neutral_ | Mean difference | Šídák's multiple comparisons test | | | |  |
|  |  |  |  |  |  |  | SE | t | DF | p |  |
| relieved | 3b | females | 1^st^ | 17,53 | 10,46 | 7,07 | 2,25 | 3,14 | 21,74 | **0,02** |  |
|  |  |  | 2^nd^ | 13,83 | 12,32 | 1,52 | 2,41 | 0,63 | 21,19 | 0,95 |  |
|  |  |  | 3^rd^ | 12,53 | 12,22 | 0,31 | 2,57 | 0,12 | 21,96 | 1,00 |  |
|  |  |  | 4^th^ | 8,95 | 9,40 | -0,45 | 2,12 | 0,21 | 20,55 | 1,00 |  |
|  |  |  |  |  |  |  |  |  |  |  |  |
|  | 3c | males | 1^st^ | 20,25 | 12,17 | 8,09 | 2,61 | 3,09 | 23,59 | **0,02** |  |
|  |  |  | 2^nd^ | 18,09 | 12,51 | 5,59 | 2,77 | 2,01 | 19,25 | 0,21 |  |
|  |  |  | 3^rd^ | 14,44 | 13,78 | 0,65 | 2,78 | 0,24 | 18,91 | 1,00 |  |
|  |  |  | 4^th^ | 15,61 | 10,97 | 4,64 | 2,84 | 1,64 | 19,79 | 0,39 |  |
|  |  |  |  |  |  |  |  |  |  |  |  |
| hungry | 3e | females | 1^st^ | 17,44 | 5,92 | 11,53 | 2,81 | 4,10 | 10,62 | **0,01** |  |
|  |  |  | 2^nd^ | 13,10 | 7,96 | 5,14 | 2,97 | 1,73 | 11,98 | 0,37 |  |
|  |  |  | 3^rd^ | 7,72 | 10,69 | -2,97 | 3,46 | 0,86 | 12,37 | 0,88 |  |
|  |  |  | 4^th^ | 13,09 | 7,55 | 5,54 | 2,84 | 1,95 | 13,92 | 0,26 |  |
|  |  |  |  |  |  |  |  |  |  |  |  |
|  | 3f | males | 1^st^ | 16,34 | 8,93 | 7,41 | 2,40 | 3,08 | 17,21 | **0,03** |  |
|  |  |  | 2^nd^ | 11,88 | 9,33 | 2,55 | 2,17 | 1,18 | 17,98 | 0,69 |  |
|  |  |  | 3^rd^ | 7,79 | 10,98 | -3,19 | 2,78 | 1,15 | 16,55 | 0,71 |  |
|  |  |  | 4^th^ | 6,72 | 7,45 | -0,73 | 2,20 | 0,33 | 17,99 | 1,00 |  |

| **Table S8. Affective state discrimination test.** Anova with repeated measures table for sniffing demonstrators % | | | | | | | | | | | | | | | | | | | | | | | |
| --- | --- | --- | --- | --- | --- | --- | --- | --- | --- | --- | --- | --- | --- | --- | --- | --- | --- | --- | --- | --- | --- | --- | --- |
| Demonstrators state | Fig. | Source of variation | | | | | | | | | | | | | | | | | | | |  | |
|  |  | Time | | | | |  | Sex | | | | | |  | | Interaction | | | | | |  | |
|  |  | SS | DF | MS | F | p |  | SS | DF | MS | F | p |  | | SS | | DF | MS | F | p |  |  |  |
| relieved | 3d | 2577,00 | 3,00 | 859,00 | 3,14 | **0,04** |  | 138,70 | 1,00 | 138,70 | 0,39 | 0,54 |  | | 521,40 | | 3,00 | 173,80 | 0,63 | 0,60 |  | |  |
|  |  |  |  |  |  |  |  |  |  |  |  |  |  | |  |  |  |  |  |  |  | |  |
|  |  |  |  |  |  |  |  |  |  |  |  |  |  | |  |  |  |  |  |  |  | |  |
|  |  |  |  |  |  |  |  |  |  |  |  |  |  | |  |  |  |  |  |  |  |  |  |
| hungry | 3g | 5341,00 | 3,00 | 1780,00 | 4,01 | **0,02** |  | 1134,00 | 1,00 | 1134,00 | 2,05 | 0,17 |  | | 784,60 | | 3,00 | 261,50 | 0,59 | 0,63 |  |  |  |
|  |  |  |  |  |  |  |  |  |  |  |  |  |  | |  |  |  |  |  |  |  | |  |
|  |  |  |  |  |  |  |  |  |  |  |  |  |  | |  |  |  |  |  |  |  | |  |
|  |  |  |  |  |  |  |  |  |  |  |  |  |  | |  |  |  |  |  |  |  | |  |

| **Table S9. Affective state discrimination test.** One sample t-test for sniffing relieved demonstrators % | | | | | | | | | | | |
| --- | --- | --- | --- | --- | --- | --- | --- | --- | --- | --- | --- |
| Demonstrators state | Fig. | Sex | Sniffing relieved/hungry demonstrators % | | | | | | | |  |
|  |  |  | n | M | SEM |  | Time [minute] | One sample t test. Comparison with hypothetical mean (50%) | | |  |
|  |  |  |  |  |  |  |  | t | df | p |  |
| relieved | 3d | females | 12 | 63,83 | 2,72 |  | 1^st^ | 5,08 | 11,00 | **0,00** |  |
|  |  |  |  | 52,25 | 4,61 |  | 2^nd^ | 0,49 | 11,00 | 0,64 |  |
|  |  |  |  | 50,00 | 4,76 |  | 3^rd^ | 0,00 | 11,00 | 1,00 |  |
|  |  |  |  | 50,33 | 6,30 |  | 4^th^ | 0,05 | 11,00 | 0,96 |  |
|  |  |  | 13 |  |  |  |  |  |  |  |  |
|  |  | males |  | 62,15 | 4,96 |  | 1^st^ | 2,45 | 13,00 | **0,03** |  |
|  |  |  |  | 57,77 | 4,265 |  | 2^nd^ | 1,82 | 13,00 | 0,09 |  |
|  |  |  |  | 47,46 | 5,629 |  | 3^rd^ | 0,45 | 13,00 | 0,66 |  |
|  |  |  |  | 58,46 | 4,635 |  | 4^th^ | 1,83 | 13,00 | 0,09 |  |
| hungry | 3g | females | 8 | 73,10 | 6,18 |  | 1^st^ | 3,74 | 7,00 | **0,01** |  |
|  |  |  |  | 65,59 | 5,32 |  | 2^nd^ | 2,93 | 7,00 | **0,02** |  |
|  |  |  |  | 46,61 | 10,05 |  | 3^rd^ | 0,34 | 7,00 | 0,75 |  |
|  |  |  |  | 64,24 | 7,33 |  | 4^th^ | 1,94 | 7,00 | 0,09 |  |
|  |  |  | 10 |  |  |  |  |  |  |  |  |
|  |  | males |  | 65,22 | 4,261 |  | 1^st^ | 3,57 | 9,00 | **0,01** |  |
|  |  |  |  | 60,97 | 5,238 |  | 2^nd^ | 2,09 | 9,00 | 0,07 |  |
|  |  |  |  | 45,80 | 9,404 |  | 3^rd^ | 0,45 | 9,00 | 0,67 |  |
|  |  |  |  | 45,59 | 7,961 |  | 4^th^ | 0,55 | 9,00 | 0,59 |  |
|  |  |  |  |  |  |  |  |  |  |  |  |

| **Table S8.** Continued. | | |  |  |  |  |  |  |  |
| --- | --- | --- | --- | --- | --- | --- | --- | --- | --- |
| Demonstrators state | Fig. | Comparison of time sniffing relieved demonstrators females vs males % | | | | | | | |
|  |  | Time [minute] | M_females_ | M_males_ | Mean difference | Šídák's multiple comparisons test | | | |
|  |  |  |  |  |  | SE | t | DF | p |
| relieved | 3d | 1^st^ | 63,83 | 62,15 | 1,68 | 5,66 | 0,30 | 18,49 | 1,00 |
|  |  | 2^nd^ | 52,25 | 57,77 | -5,52 | 6,28 | 0,88 | 22,66 | 0,86 |
|  |  | 3^rd^ | 50,00 | 47,46 | 2,54 | 7,37 | 0,34 | 22,66 | 1,00 |
|  |  | 4^th^ | 50,33 | 58,46 | -8,13 | 7,82 | 1,04 | 20,59 | 0,77 |
| hungry | 3g | 1^st^ | 73,10 | 65,22 | 7,88 | 7,51 | 1,05 | 12,96 | 0,91 |
|  |  | 2^nd^ | 65,59 | 60,97 | 4,62 | 7,47 | 0,62 | 15,69 | 0,99 |
|  |  | 3^rd^ | 46,61 | 45,80 | 0,81 | 13,77 | 0,06 | 15,42 | 1,00 |
|  |  | 4^th^ | 64,24 | 45,59 | 18,65 | 10,82 | 1,72 | 15,97 | 0,27 |

| **Table S10. Affective state discrimination test.** One sample t-test for sniffing relieved demonstrators %. | | | | | | | | | | | | | | | | | | | | | | | | | | |
| --- | --- | --- | --- | --- | --- | --- | --- | --- | --- | --- | --- | --- | --- | --- | --- | --- | --- | --- | --- | --- | --- | --- | --- | --- | --- | --- |
| Demonstrators position (east vs west) comparison. | | | | | | | | | |  | |  |  |  |  | |  | |  | |  | |  | |  | |
| Demonstrators state | Fig. | Sex | Demonstrator's position | Sniffing relieved/hungry demonstrators % | | | | | | | | | | | | | | | | | | | | |  | |
|  |  |  |  | n | M | SEM |  | One sample t test. Comparison with hypothetical mean (50%) | | | | |  | Mean difference |  | Unpaired Student’s t-test | | | | | |  | |  | |  |
|  |  |  |  |  |  |  |  | t | df | | p | |  |  |  | t | | df | | p | |  | |  | |  |
| relieved | S2a | females | west | 7 | 64,14 | 3,47 |  | 4,07 | 6,00 | | **0,01** | |  | 2,14 |  | 0,39 | | 10,00 | | 0,70 | |  | |  | |  |
|  |  |  | east | 5 | 62,00 | 4,23 |  | 2,84 | 4,00 | | **0,05** | |  |  |  |  |  |  |  |  |  |  | |  | |  |
|  | S2b | males | west | 8 | 65,00 | 6,43 |  | 2,33 | 7,00 | | 0,05 | |  | 8,20 |  | 0,78 | | 11,00 | | 0,45 | |  | |  | |  |
|  |  |  | east | 5 | 56,80 | 8,49 |  | 0,80 | 4,00 | | 0,47 | |  |  |  |  |  |  |  |  |  |  | |  | |  |
| hungry | S2f | females | west | 4 | 67,76 | 9,97 |  | 1,78 | 3,00 | | 0,17 | |  | 10,67 |  | 0,85 | | 6,00 | | 0,43 | |  | |  | |  |
|  |  |  | east | 4 | 78,43 | 7,74 |  | 3,67 | 3,00 | | **0,03** | |  |  |  |  |  |  |  |  |  |  | |  | |  |
|  | S2g | males | west | 4 | 68,24 | 9,20 |  | 1,98 | 3,00 | | 0,14 | |  | 5,03 |  | 0,56 | | 8,00 | | 0,59 | |  | |  | |  |
|  |  |  | east | 6 | 63,21 | 4,32 |  | 3,06 | 5,00 | | **0,03** | |  |  |  |  |  |  |  |  |  |  | |  | |  |

| **Table S11. Affective state discrimination test.** One sample t-test for sniffing relieved/hungry to be demonstrators %. | | | | | | | | | | | | | | | | | | | | | | | | | |
| --- | --- | --- | --- | --- | --- | --- | --- | --- | --- | --- | --- | --- | --- | --- | --- | --- | --- | --- | --- | --- | --- | --- | --- | --- | --- |
| Demonstrators state | Fig. | Sex | Sniffing relieved/hungry to be demonstrators % | | | | | | | | | | | | | | |  | |  | |  | |  | |
|  |  |  | n | M | SEM |  | One sample t test. Comparison with hypothetical mean (50%) | | |  | Mean difference |  | Unpaired Student’s t-test | | |  | |  |  | |  | |  | |  |
|  |  |  |  |  |  |  | t | df | p |  |  |  | t | df | p | |  |  |  | |  | |  | |  |
| relieved | S2e | females | 9 | 35,44 | 5,83 |  | 2,50 | 8,00 | **0,04** |  | 10,06 |  | 1,28 | 19,00 | 0,22 | |  |  |  | |  | |  | |  |
|  |  | males | 12 | 45,50 | 5,19 |  | 0,87 | 11,00 | 0,40 |  |  |  |  |  |  |  |  |  |  | |  | |  | |  |
| hungry | S2h | females | 8 | 59,32 | 3,98 |  | 2,34 | 7,00 | 0,05 |  | 20,46 |  | 2,01 | 16,00 | 0,06 | |  |  |  | |  | |  | |  |
|  |  | males | 10 | 38,86 | 8,50 |  | 1,31 | 9,00 | 0,22 |  |  |  |  |  |  |  |  |  |  | |  | |  | |  |

| **Table S12.** Groups of animals used in Figures 1-3. Only mice that met predefined criteria. | | | | | | | | | | | | | | |  |
| --- | --- | --- | --- | --- | --- | --- | --- | --- | --- | --- | --- | --- | --- | --- | --- |
| Sex | Behavioral test | experiment variant | n | Subject's age at the start of the procedure (weeks)^a^ | | |  | Subject's age at test (weeks)^b^ | | |  | Subject's weight at the start of the procedure [g]^a^ | | | |
|  |  |  |  | M | Range | SEM |  | M | Range | SEM |  | M | Range | SEM | |
| females | Prosocial choice test**^1^** |  | 8 | 10,30 | 9,90-10,40 | 0,06 |  | 11,70 | 11,30-11,90 | 0,07 |  | 19,85 | 18,60-21,00 | 0,28 | |
|  | Affective state discrimination test**^2^** | relieved | 12 | 12,62 | 10,90-14,60 | 0,31 |  | 13,09 | 11,30-15,90 | 0,36 |  | 20,38 | 18,50-22,20 | 0,37 | |
|  |  | hungry | 8 | 11,49 | 9,90-13,40 | 0,52 |  | 11,94 | 10,30-13,90 | 0,53 |  | 21,28 | 19,40-23,20 | 0,41 | |
|  | Social conditioned place preference test**^3^** |  | 16 | 10,73 | 10,60-10,90 | 0,03 |  | 11,56 | 11,40-11,90 | 0,05 |  | 18,69 | 17,40-19,60 | 0,28 | |
| males | Prosocial choice test**^1^** |  | 10 | 10,30 | 9,90-11,00 | 0,14 |  | 11,70 | 11,00-12,40 | 0,15 |  | 26,65 | 24,10-28,60 | 0,45 | |
|  | Affective state discrimination test**^2^** | relieved | 13 | 11,40 | 9,90-12,60 | 0,24 |  | 11,95 | 10,30-14,00 | 0,30 |  | 26,05 | 23,30-29,10 | 0,49 | |
|  |  | hungry | 10 | 11,52 | 10,00-14,10 | 0,43 |  | 12,07 | 10,70-14,60 | 0,43 |  | 26,69 | 24,80-31,00 | 0,58 | |
|  | Social conditioned place preference test**^3^** |  | 12 | 10,60 | 10,00-11,00 | 0,14 |  | 11,60 | 11,00-12,00 | 0,14 |  | 23,90 | 21,30-26,00 | 0,40 | |

| **Table S12.** Continued. | |  |  |  |  |  |  |  |  |  |  |  |  |  |  |
| --- | --- | --- | --- | --- | --- | --- | --- | --- | --- | --- | --- | --- | --- | --- | --- |
| Sex | Behavioral test | Experiment variant | n | Subject's weight at test [g]^b^ | | |  | Stimulus' weight at the start of the procedure [g]^c^ | | |  | Stimulus' weight at test [g]^d^ | | |  |
|  |  |  |  | M | Range | SEM |  | M | Range | SEM |  | M | Range | SEM |  |
| females | Prosocial choice test**^1^** |  | 8 | 17,01 | 15,80-18,40 | 0,34 |  | 18,25 | 16,70-19,90 | 0,34 |  | 15,50 | 13,8-16,90 | 0,34 |  |
|  | Affective state discrimination test**^2^** | relieved | 12 | 19,83 | 17,20-21,50 | 0,62 |  | 19,68 | 17,40-21,30 | 0,42 |  | 16,53 | 15,40-18,70 | 0,49 |  |
|  |  | hungry | 8 | 21,70 | 21,20-22,50 | 0,22 |  | 20,65 | 19,40-21,60 | 0,25 |  | 17,68 | 17,20-18,30 | 0,20 |  |
|  | Social conditioned place preference test**^3^** |  | 16 | 19,82 | 17,40-21,50 | 0,31 |  | NA | NA | NA |  | NA | NA | NA |  |
| males | Prosocial choice test**^1^** |  | 10 | 22,81 | 20,60-25,40 | 0,47 |  | 25,24 | 20,60-25,40 | 0,60 |  | 21,40 | 22,90-27,40 | 0,45 |  |
|  | Affective state discrimination test**^2^** | relieved | 13 | 25,42 | 23,20-28,10 | 0,51 |  | 24,65 | 21,00-28,20 | 0,57 |  | 20,95 | 17,30-26,80 | 0,73 |  |
|  |  | hungry | 10 | 27,23 | 24,80-30,20 | 0,74 |  | 25,21 | 23,40-28-60 | 0,49 |  | 21,56 | 19,90-23,10 | 0,65 |  |
|  | Social conditioned place preference test**^3^** |  | 12 | 23,74 | 21,60-25,50 | 0,34 |  | NA | NA | NA |  | NA | NA | NA |  |
| \| **Table S13. Social conditioned place preference test.** Conditioning cues. \| \| \| \| \|  \| \| --- \| --- \| --- \| --- \| --- \| --- \| \|  \| Name \| Full name \| Description/size [mm] \| Manufacterer \| Website \| \| Bedding types \| Aspen 1 \| ABEDD aspen animal bedding \| cubic granulates \| Abedd SIA Jelgavas iela 29 Kalnciems, LV-3016, Latvia \| <https://www.abedd.com/> \| \| Beech 1 \| Trociny bukowe przesiane gat. 1 \| shavings \| P.P.H. "WO-JAR", Kopernika 3/30, 32-100 Proszowice, Poland \| NA \| \| Beech 2 \| Trocinka bukowa Facimiech \| shavings \| PPHU Natur-Drew A. Czaja, os. Kopernika 5/57, 34-100 Wadowice, Poland \| NA \| \| Cellulose \| Biofresh Performance Bedding. 1/8' Pelleted Cellulose \| pellets \| ABSORPTION CORP 6960 Salashan Parkway Ferndale, WA 98248, USA \| <https://scottpharma.net/product/biofresh-performance-bedding/> \| \| Gnawing block types \| Block 1 \| Long thin gnawing block \| 99 × 19 × 19 \| Urszula Borgiasz Zoolab, Zielona 14, 28-340 Sedziszow, Poland \| <http://zoolab.pl/en/enrichment-elements/> \| \| Block 2 \| Long big gnawing block ("for rats"). \| 99 × 39 × 39 \| \| Block 3 \| Cube gnawing block \| 49 × 39 × 39 \|   **1a.** Actor's age/weight at start of the food deprivation; **1b.** Actor's age/weight on the first day of test; **1c.** Partner's weight on the start of the food deprivation; **1d.** Partner's weight on the first day of test. **2a**. Observer's age/weight on the first day of adaptation; **2b**. Observer's age/weight on test day; **2c**. Relieved/hungry demonstrator's weight on the first day of adaptation. 3a. Subject's age/weight on the day of pretest; **3b**. Subject's age/weight on the day of posttest. | | | | | | | | | | | | | | |  |
|  |  |  |  |  |  |  |  |  |  |  |  |  |  |  |  |
|  |  |  |  |  |  |  |  |  |  |  |  |  |  |  |  |
|  |  |  |  |  |  |  |  |  |  |  |  |  |  |  |  |

| **Table S14. Social conditioned place preference test.** Number of mice excluded based on predefined criteria**.** | | | | | | | | | | | | | |
| --- | --- | --- | --- | --- | --- | --- | --- | --- | --- | --- | --- | --- | --- |
| sCPP protocol | Sex | Initial n | Number of mice that did not meet criterion A | Tested n |  |  |  |  |  |  |  |  |  |
| 6 days | females | 17 | 1 | 16 |  |  |  |  |  |  |  |  |  |
| 6 days | males | 12 | 0 | 12 |  |  |  |  |  |  |  |  |  |
| 2 days | males | 8 | 0 | 8 |  |  |  |  |  |  |  |  |  |
| Criterion A: Initial preference to any of the context not exceeding 70% in pretest. | | | |  |  |  |  |  |  |  |  |  |  |

| **Table S15. Affective state discrimination test.** One sample t-test for sniffing relieved demonstrators %. | | | | | | | | | | | | | | | | | | | |
| --- | --- | --- | --- | --- | --- | --- | --- | --- | --- | --- | --- | --- | --- | --- | --- | --- | --- | --- | --- |
| Demonstrators position (cups vs no cups) comparison. | | | | | | | | | | |  |  |  |  |  |  |  |  |  |
| Demonstrators state | Fig. | Sex | Demonstrator's position | Sniffing relieved/hungry demonstrators % | | | | | | | | | | | | | |  | |
|  |  |  |  | n | | M | SEM |  | One sample t test. Comparison with hypothetical mean (50%) | | |  | Mean difference |  | Unpaired Student’s t-test | | |  |  |
|  |  |  |  |  |  |  |  |  | t | df | p |  |  |  | t | df | p |  |  |
| relieved | S2c | females | cups | | 7 | 62,57 | 3,75 |  | 3,35 | 6,00 | **0,02** |  | 1,63 |  | 0,30 | 10,00 | 0,77 |  |  |
|  |  |  | no cups | | 5 | 64,20 | 3,73 |  | 3,80 | 4,00 | **0,02** |  |  |  |  |  |  |  |  |
|  | S2d | males | cups | | 7 | 62,00 | 7,62 |  | 1,57 | 6,00 | 0,17 |  | 0,33 |  | 0,03 | 11,00 | 0,98 |  |  |
|  |  |  | no cups | | 6 | 61,67 | 7,13 |  | 1,64 | 5,00 | 0,16 |  |  |  |  |  |  |  |  |
| In the experiments with hungry demonstrators during the first day of habituation there were always cups in test cage. | | | | | | | | | | | | | |  |  |  |  |  |  |

| **Table S16. Affective state discrimination test.** Number of mice excluded based on predefined criteria**.** | | | | | | | | | |
| --- | --- | --- | --- | --- | --- | --- | --- | --- | --- |
| Demonstrators state | Sex | Initial n | Number of mice that did not meet criterion A | Tested n |  |  |  |  |  |
| relieved | females | 14 | 2 | 12 |  |  |  |  |  |
|  | males | 15 | 2 | 13 |  |  |  |  |  |
| hungry | females | 9 | 1 | 8 |  |  |  |  |  |
|  | males | 11 | 1 | 10 |  |  |  |  |  |
|  |  |  |  |  |  |  |  |  |  |
